# Neuroactive Steroids as Novel Promising Drugs in Therapy of Postpartum Depression—Focus on Zuranolone

**DOI:** 10.3390/ijms26136545

**Published:** 2025-07-07

**Authors:** Jolanta B. Zawilska, Ewa Zwierzyńska

**Affiliations:** Department of Pharmacodynamics, Medical University of Łódź, Muszyńskiego 1, 90-151 Łódź, Poland; ewa.zwierzynska@umed.lodz.pl

**Keywords:** antidepressants, GABA, neuroactive steroids, allopregnanolone, postpartum depression, pharmacotherapy, brexanolone, zuranolone

## Abstract

Postpartum depression (PPD) remains a significant health concern worldwide. Both non-pharmacological and pharmacological treatments are available for patients with PPD; however, the standard approach involving selective serotonin reuptake inhibitors (SSRIs) and other antidepressants fails to provide a rapid response. This narrative review presents basic clinical and epidemiological data on PPD, summarizes currently used pharmacotherapies of PPD, highlights their limitations, and discusses new therapies based on a revised understanding of the disease’s pathogenesis. Numerous studies indicate that dysregulation of GABAergic neurotransmission, which may result from fluctuating levels of neuroactive steroids during pregnancy and the postpartum period, plays an important role in the complex pathology of PPD. Considering this, neuroactive steroids, which act as positive allosteric modulators of central GABA_A_ receptors (GABA_A_Rs), may offer new promising avenues for treating PPD. The first rapid-acting neurosteroid approved by the FDA to treat PPD in women is brexanolone, although its use is constrained by pharmacokinetic properties. The first oral neuroactive steroid-based antidepressant approved by the FDA for PPD is zuranolone. This review discusses the molecular mechanism of zuranolone action and the results of preclinical and clinical studies regarding the effectiveness and safety of the drug in treating PPD.

## 1. Postpartum Depression

Depressive disorders are considered to be among the most common mental illnesses worldwide [1]. One of the “faces of depression” is postpartum depression (PPD), also known as perinatal depression. Low mood and apathy right after giving birth are the norm. This phenomenon, called “baby blues” (or postpartum blues), affects 50% to 80% of mothers. Postpartum blues tends to begin 1 to 3 days after parturition, lasting at least 2 weeks, and sometimes continues to develop into PPD [2]. According to the Diagnostic and Statistical Manual of Mental Disorders—Fifth Edition (DSM-5), PPD is an episode of a major depressive disorder (MDD) [3]. For PPD diagnosis the 10-item Edinburgh Postnatal Depression Scale (EPDS), endorsed by the American Academy of Pediatrics and the American College of Obstetricians and Gynecologists is also used [4]. Guidelines and definitions of PPD vary with respect to the time period of onset, and usually include symptoms starting during pregnancy or within the period from 4 weeks to 12 months postpartum [3,4]. The peaks of PPD occur during the first 6 months postpartum, especially at 2–3 weeks and 6–8 weeks [5]. PPD is a global health issue—it can affect women from all races, ethnicities, cultures, and educational or economic backgrounds. PPD is one of the most prevalent psychiatric disorders during the peripartum period [6]. The prevalence of PPD varies substantially depending on the definition of the disorder, country, diagnostic tools used, threshold of discrimination chosen for the screening measure, and period over which the prevalence is determined. In a recent systematic review, Bai and coworkers assessed the prevalence of PPD based exclusively on studies using diagnostic interviews. The pooled prevalence of all forms of depression and major depression within one year postpartum was 12.1% and 7.0%, respectively [5]. Despite having a high prevalence, PPD is underdiagnosed and undertreated [7]. Risk factors for PPD include a history of depression, a history of physical or sexual abuse, carrying an unplanned or unwanted pregnancy, younger age, multiparity, obstetric difficulties, poor marital relationship, being unemployed, poor sleep quality, lack of social support, and congenital abnormality [8,9,10,11,12,13,14,15].

PPD is characterized by a mix of symptoms, like low mood. The woman often cries, even for no reason; she is irritated, sad, and depressed for most of the day; she has a lack of energy to perform even basic activities; she shows a loss of interests, anhedonia, lowered self-esteem, and a sense of worthlessness (e.g., thinking about herself, “I am a bad, hopeless mother”, “I am not cut out to be a mother”); she has an excessive or inadequate sense of guilt (e.g., “I do not take care of the child as I should”, “I do not devote myself enough to the child, therefore I am a bad mother”, “I think too much about myself and too little about the child”); she feels a sense of helplessness and inability to cope in the role of a mother (e.g., “Taking care of a child is beyond me”); and she suffers from insomnia, cognitive disturbances, impaired concentration/decision making, memory problems, and anxiety states related to, among other things, often unfounded worries about the child’s health, as well as the need to care for it. Anxiety may be present in approximately 70% of women with PPD [6,16]. Severe PPD symptomatology is correlated with suicidal ideation and significantly increases the risk of completed suicide [17,18]. When compared to MDD, PPD exhibits several distinct characteristics, i.e., the onset of the disease, and more anxiety, psychomotor symptoms, obsessive thoughts, impaired concentration, fatigue, and loss of energy, but less psychoticism, sleep disturbances, and suicidal ideation [6]. PPD is associated with numerous short- and long-term outcomes for the mother, the child, and the entire family, especially if it remains undetected and untreated [19,20,21]. In severe cases, PPD can lead to neglect of the infant or thoughts of harming the infant [22,23]. The disease is associated with poor maternal functioning, worse quality of the mother’s life, poor physical and mental health of the mother, disruption of mother–infant bonding, and relational harms in the mother–child–partner triad. Several studies demonstrate that PPD may have a negative impact on the emotional, cognitive, and social development of a child [24,25,26,27,28,29]. An estimated mean time for a full remission of PPD is 49 weeks, with 30% of patients recovering at 6 months, 66% at 12 months, and 94% at 24-month follow-up [30]. Analysis of longitudinal clinical studies on PPD indicated that at any time point between 4 months and 3 years postpartum, about 30% of mothers diagnosed with PPD still meet the criteria for depression [31].

PPD is a very heterogeneous disorder, and various biological, psychological, and sociocultural factors underlie the etiology of the disease. The pathophysiology of PPD involves a complex interplay between different processes. Despite numerous studies, our understanding of the pathogenesis of PPD still remains incomplete. Recent hypotheses suggest that multiple neurobiological factors, such as hormonal changes, including dysregulation of the hypothalamic–pituitary–gonadal and hypothalamic–pituitary–adrenal axes, neurotransmitter imbalances, disturbances in functioning of neural networks, neuroactive steroids, psychosocial stressors, and inflammation, all play important roles in the pathophysiology of PPD [32].

## 2. Current Therapeutic Approaches for PPD

There are two basic approaches to treat PPD: non-pharmacological treatment and pharmacotherapy. Non-pharmacological treatments include cognitive and behavioral psychotherapy, psychodynamic psychotherapy, psychoeducation, psychosocial interventions, supportive counseling, interpersonal psychotherapy, creative art therapy, and light therapy. Other non-pharmacological treatments for PPD, like electroconvulsive therapy and repetitive transcranial magnetic stimulation, are also used, but much less often [33,34,35,36,37]. Of these, cognitive behavioral psychotherapy and interpersonal therapy are frequently recommended as first-line treatments for mild-to-moderate PPD [36].

For decades, pharmacological treatment strategies for depression have been based primarily on the monoaminergic deficiency hypothesis. This theory assumes that depression is caused by an imbalance between monoaminergic neurotransmitters in the brain, mainly serotonin and noradrenaline, or their pathologically low levels. According to this hypothesis, the therapeutic effects of antidepressants, including, among others, tricyclic antidepressants (TCAs), selective serotonin reuptake inhibitors (SSRIs), and serotonin and noradrenaline reuptake inhibitors (SNRIs), result from increased serotoninergic/noradrenergic transmission in the brain [38]. However, the currently used antidepressants have three important limitations: (1) although synaptic monoamine concentrations increase rapidly after taking these drugs, the therapeutic effects occur with a long delay of several weeks; (2) about 30% of patients suffer from treatment-resistant depression; and (3) the drugs exert several adverse effects, including, among others, weight gain, sexual dysfunction, cardiovascular disturbances, and sleep problems [38].

Historically, the gold standard pharmacologic treatment for PPD has been focused on using antidepressants indicated for MDD outside of the perinatal period, with SSRIs being the most common. However, classical antidepressants do not offer a rapid treatment response, nor are they specific for PPD. In the United States, conventional antidepressants are not approved by the Food and Drug Administration (FDA) for the treatment of PPD (Table 1), thus their use is considered off-label (for reviews see [39,40,41,42]). The facts presented above clearly indicate that there is an urgent need for novel rapidly acting, specific therapies for PPD. One promising avenue to achieve this goal is based on neuroactive steroids and the gamma aminobutyric acid (GABA) signaling hypothesis. According to this hypothesis, dysfunctional GABAergic signaling related to stress, fluctuations in reproductive hormone levels, and rapid loss of circulating levels of allopregnanolone, a neuroactive steroid, at parturition, may play an important role in the pathophysiology of PPD [43].

## 3. In Search of New Drugs for PPD—Neuroactive Steroids and GABAergic Signaling Hypothesis

Functional communication within and between brain circuits is tightly controlled by a balance between excitatory (glutamatergic) and inhibitory (GABAergic) inputs. Depression is associated with dysregulated excitation–inhibition balance in part due to decreased GABA levels or signaling activity. GABA exerts biological effects by stimulating specific membrane bound receptors, namely ionotropic GABA_A_Rs and metabotropic G-protein coupled GABA_B_Rs. GABA_A_Rs are heteropentamers composed of various subunits, typically a combination of α1-6, β1-4, γ1-3, δ, ε, θ, and ρ1-3, that form ligand-gated chloride channels. Binding of drugs to GABA_A_R depends on its subunit composition [49]. When activated, the channels open, permitting chloride ion influx, and thus hyperpolarization of the cell membrane. GABA can elicit CNS inhibition as either a short signal in response to activation of synaptic GABA_A_Rs (phasic inhibition), or a longer-lasting inhibition in response to activation of extrasynaptic GABA_A_Rs (tonic inhibition). Tonic inhibition regulates excitation through long-term hyperpolarization and plays an important role in synaptic plasticity, neurogenesis, and cognitive functions [50].

**Figure 1:**
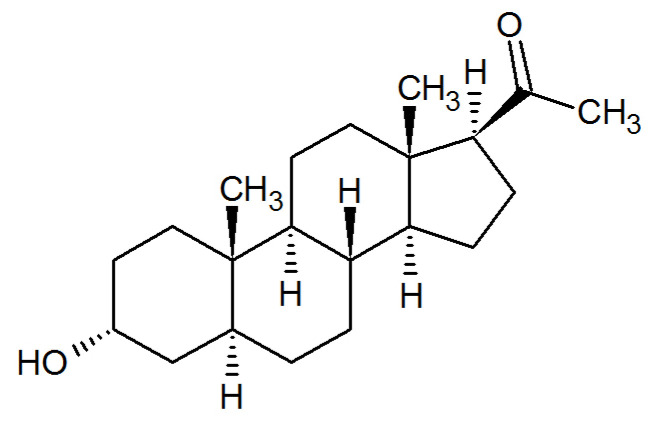
The chemical structure of allopregnanolone.

Steroid hormones play a critical role during pregnancy, and of particular importance is a progesterone metabolite and neuroactive steroid, allopregnanolone (3α-hydroxy-5α-9pregnan-20-one) (Figure 1). Allopregnanolone is generated from progesterone by sequential actions of 5α-reductase and 3α-hydroxysteroid dehydrogenase. Levels of allopregnanolone increase during pregnancy, decline after birth, and remain low up to 6 months postpartum [51,52]. Concomitant with an increase in allopregnanolone levels during pregnancy, a decrease in GABA_A_R expression occurs, yielding a stable inhibition. Following a rapid decline of allopregnanolone at parturition, the recovery of GABA_A_R expression is delayed, creating a hyperexcitable state that may persist and participate in the symptomology of PPD [40,51,53].

Neuroactive steroids are endogenous neuromodulators that rapidly alter neuronal excitability by binding to membrane-bound receptors. They can be de novo synthesized in the brain from cholesterol, particularly within excitatory neurons, but also glial cells, and their synthesis is altered by acute cellular and environmental stressors [54,55]. They can also reach the brain from peripheral steroidogenic organs, such as the adrenal glands and gonads [56]. A major class of these steroids includes allopregnanolone, a progesterone metabolite [57]. Neuroactive steroids powerfully modulate major targets in the brain by affecting ligand-gated and voltage-gated ion channels that regulate neuronal excitability, intercellular communication, and plasticity [58]. They are essential in the regulation of the hypothalamic–pituitary–adrenal axis during acute and chronic stress [59,60]. In addition, they exert anti-inflammatory and neurotrophic effects [59,61,62,63], and induce potent anxiolytic, antidepressant, anticonvulsant, sedative, analgesic, and amnesic effects, mainly through interaction with the GABA_A_Rs in the brain [56].

**Figure 2:**
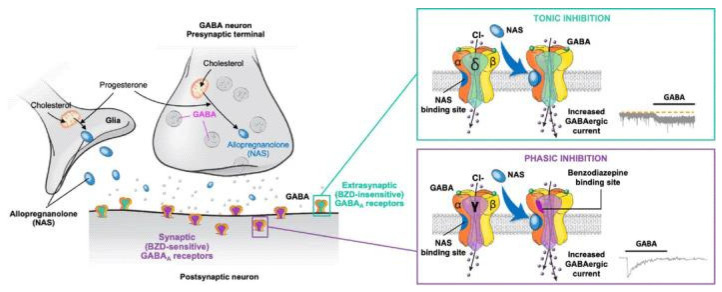
Mechanism of action of the positive allosteric modulator allopregnanolone on synaptic and extrasynaptic GABA_A_ receptors. BZD, benzodiazepine; GABA, γ-aminobutyric acid; NAS, neuroactive steroid. Figure is reproduced from Gunduz-Bruce et al., 2022 [66] according to the terms of the Creative Commons Attribution License.

Neuroactive steroids are positive allosteric modulators (PAMs) of GABA_A_Rs in the brain. They prolong the opening time of chloride ion channels within GABA_A_Rs, thus enhancing inhibitory neurotransmission. It is hypothesized that neuroactive steroids restore excitation–inhibition balance in the brain areas dysregulated in depression [64]. Although they bind to GABA_A_Rs containing δ, α4, or α6 subunits localized both at synaptic and extrasynaptic sites, they preferentially interact with extrasynaptic GABA_A_Rs containing δ subunits, and at lower concentrations specifically enhance a tonic inhibitory conductance that is mediated by this type of receptors. At higher concentrations, neuroactive steroids also potentiate phasic inhibition mediated by synaptic α1β2γ2 GABA_A_Rs [65,66,67]. In contrast, benzodiazepines, another group of positive allosteric modulators of GABA_A_Rs, primarily activate synaptic GABA_A_R receptors by binding to the interface of the ɣ2 subunit and α1-3 and α5 subunits; GABA_A_Rs with this composition are localized to synaptic sites (Figure 2) [68].

The distinct sites of neuroactive steroid action at GABA_A_Rs, when compared with other PAMs, offer a new opportunity to explore a GABA-based treatment approach for PPD [69,70]. In line with this, recent studies performed on mice demonstrated that allopregnanolone, but not diazepam (a benzodiazepine drug), increased theta oscillation in the basolateral amygdala and medial prefrontal cortex by stimulating δ-subunit-containing GABA_A_Rs. Furthermore, only allopregnanolone exhibited antidepressant-like effects in chronic unpredictable stress and social defeat stress mouse models of depression [55,69,70].

## 4. Neuroactive Steroids as Novel Promising Drugs in Therapy of PPD

### 4.1. Brexanolone

On 19 March 2019, brexanolone (SAGE-547; ZULRESSO™, Sage Therapeutics, Inc, Cambridge, MA, USA) became the first rapid-acting neuroactive steroid drug approved by the FDA (SAGE-547; ZULRESSO™) specifically to treat PPD in women aged 18–45 years [44]. It was developed by Sage Therapeutics Inc. under a license to the University of California [71]. Brexanolone is prepared as an isotonic solution of 5 mg/mL allopreganolone buffered in 250 mg/mL sulfobutylether-β-cyclodextrin, a solubilizing agent. As brexanolone has poor bioavailability (<5%) when given orally, it is administered as a continuous intravenous infusion over 60 h (2.5 days). During infusion, patients must be accompanied by their infants. The optimal pattern of brexanolone dosing in order to maintain effective levels and cause PPD remission is intravenous administration of 30 μg/kg/h during the first 4 h; this is then increased to 60 μg/kg/h (4 to 24 h) and 90 μg/kg/h (24 to 52 h); decreased to 60 μg/kg/h (52 to 56 h); and finally decreased to 30 μg/kg/h (56 to 60 h) [72]. Three positive randomized, double-blind, placebo-controlled trials of brexanolone versus placebo demonstrated a sustained response to brexanolone infusion through 30 days of follow-up post treatment [73,74]. Significant improvements in depressive symptoms on the Hamilton Depression Rating Scale (HAMD), consistent with effects of other antidepressants but remarkably faster (60 h versus 4 weeks), were reported [75]. In humans, brexanolone is metabolized by non-CYP pathways, mainly ketoreduction, glucuronidation, and sulfation, to inactive metabolites; thus, it is not likely to be a substrate of pharmacokinetic interactions with a concomitant drug used [75]. The most common adverse effects of brexanolone are dizziness, sedation/somnolence, headaches, and dry mouth (xerostomia). Suicidal ideation, presyncope, vertigo, tachycardia, and hot flashes were also reported, but at a very low rate [73,74]. Due to the risk of serious harm in patients treated with brexanolone, during infusion monitoring for excessive sedation and sudden loss of consciousness, continuous pulse oximetry is required. At present, treatment with brexanolone is available only through the FDA-approved restricted Risk Evaluation and Mitigation Strategy (REMS) program [73,75].

### 4.2. Zuranolone (ZURZUVAE™)—The First FDA-Approved Oral Neuroactive Steroid-Based Antidepressant for PPD

**Figure 3:**
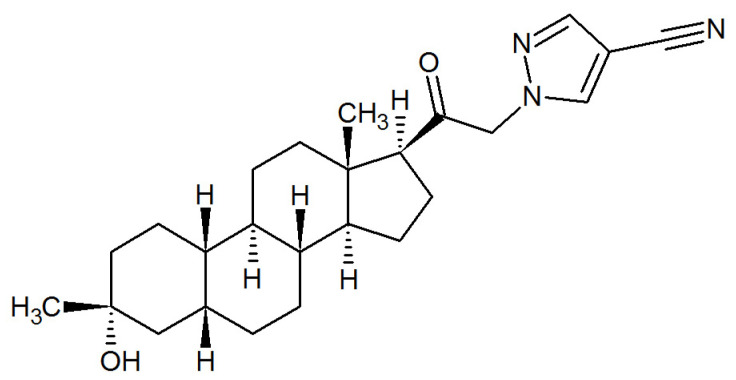
The chemical structure of zuranolone.

On 4 August 2023, the FDA approved zuranolone (SAGE-217; ZURZUVAE™), developed by Sage Therapeutics Inc. (Cambridge, MA, USA) and Biogen Inc., (Cambridge, MA, USA) as the first oral neuroactive steroid-based drug for the treatment of PPD [45,76]. The chemical structure of zuranolone, 1-[(3α,5β)-3-hydroxy-3-methyl-20-oxo-19-norpregnan-21-yl]-1H-pyrazole-4-carbonitrile, is distinct from brexanolone due to the presence of a cyanopyrazole ring at carbon 21 [77] (Figure 3). When compared with brexanolone, zuranolone has the advantage of being orally bioavailable, and thus offers the ease of once-daily, evening dosing at home.

#### 4.2.1. Zuranolone—Preclinical Studies

The effects of zuranolone on GABA_A_R were examined in vitro using a patch clamp technique. Experiments were performed on recombinant cell lines stably expressing specific receptor subunit combinations: Xenopus oocytes (α1β1γ2 and α6β3δ) and Ltk (α1β2γ2 and α2β2γ2), or Chinese hamster ovary cells transiently expressing α4β3δ. Zuranolone enhanced GABA_A_R current at α1β2γ2 receptors, with a calculated EC_50_ of 430 nM and maximum efficacy (E_max_) of 1037%, while at α4β3δ receptors with an EC_50_ of 118 nM and E_max_ of 556%. Studies conducted on hippocampal slices from postnatal day-21 mice demonstrated that zuranolone enhances both tonic and phasic conductance of GABA_A_Rs [78].

The pharmacokinetic parameters of zuranolone (10 mg/kg) were evaluated in mice after a single dose of 10 mg/kg given intraperitoneally (IP) or per os (PO). At both routes of administration, a maximum plasma concentration (C_max_) was observed after 30 min. Oral administration resulted in a lower plasma C_max_ (1335 ng/mL) compared to IP one (3197 ng/mL). After oral and IP administration, the bioavailability of zuranolone was 62% and 89%, respectively. The brain–plasma ratio following both administrations was in the range of 1.4–1.6 [78].

Pharmacological activity of zuranolone was analyzed using two animal models: pentylenetetrazol-induced seizures in mice and pharmacoEEG in rats. Zuranolone at doses of 1, 3, and 10 mg/kg given PO one hour before systemic administration of pentylenetetrazol (an antagonist of GABA_A_Rs) significantly increased the latency to tonic seizures (≥1688 s) compared to vehicle (751 s). The β-band EEG power (power in the 13–30 Hz frequency range) is a robust in vivo, translatable biomarker of GABA_A_R PAM activity. Oral administration of zuranolone (3 and 20 mg/kg) resulted in a rapid increase in electroencephalogram β-frequency power in rats [78].

#### 4.2.2. Zuranolone—Clinical Studies

##### Pharmacokinetic Parameters of Zuranolone in Humans

In adults, the mean apparent clearance (CL/F) of zuranolone was 33 L/h and terminal half-life time (t_1/2_) was 19.7 to 24.6 hrs. The mean blood-to-plasma concentration ratio ranged from 0.54 to 0.58. Zuranolone has a high binding affinity to plasma proteins >99.5%. In healthy subjects, zuranolone administered once daily at a dose of 30 mg reached a steady state after 3–5 days. It was demonstrated that a fat content of a meal has a significant impact on both the C_max_ and AUC values. Following administration of 30 mg of zuranolone with a low-fat meal (25% fat), C_max_ and AUC_last_ increased by 3.5-fold and 1.8-fold, respectively, compared to fasted conditions. In the case of a high-fat meal (50% fat), C_max_ and AUC_last_ increased by 4.3-fold and 2-fold, respectively. The t_max_ (time to reach maximum concentration) value was not affected by the fat content in the meal. Following oral administration of radiolabeled zuranolone, 45% of the dose was recovered in urine as metabolites with negligible unchanged zuranolone, and 41% in feces as metabolites with less than 2% as unchanged zuranolone [77].

Phase 1, double-blind, placebo-controlled, single ascending dose (SAD) and multiple ascending dose (MAD) studies were conducted to assess the pharmacokinetic properties of zuranolone. A total of 108 healthy volunteers were enrolled in the studies: 72 subjects in the SAD study and 36 subjects in the MAD study. In the SAD study, subjects received a single dose of zuranolone (0.25, 0.75, 2, 5.5, 11, 22, 44, 55, or 66 mg). In the MAD study, subjects received zuranolone at doses of 15 mg, 35 mg, or 30 mg in the morning for 7 days; control groups received placebo for 7 days. Following single doses of zuranolone, plasma concentrations rapidly increased and reached C_max_ approximately one hour post dose. Concentrations of the drug then declined in a biphasic manner and exhibited t_1/2_ ranging from 16 to 23 hrs. For both single and repeated dose administration, t_1/2_ and t_max_ showed dose independence [79].

Zuranolone is extensively metabolized in the liver by the CYP3A4 enzyme. This property of zuranolone indicates a possibility of serious pharmacokinetic interactions with other drugs that affect the activity of CYP3A4. Concomitant use of zuranolone with CYP3A4 inhibitors may increase the risk of its adverse effects, while use with CYP3A4 inducers may decrease the efficacy of zuranolone. Thus, it is recommended that when taking zuranolone concurrently with CYP3A4 inhibitors, the dosage must be adjusted, while usage of CYP3A4 inducers together with zuranolone should be avoided [80].

##### Therapeutical Effects and Safety of Zuranolone

A multicenter ROBIN study (217-PPD-201) was the first phase 3 randomized, double-blind, placebo-controlled study evaluating the efficacy, safety, and pharmacokinetic parameters of zuranolone (30 mg) in the treatment of women with PPD. The 17-item Hamilton Rating Scale for Depression (HAMD_17_) scale and the Montgomery–Åsberg Depression Rating Scale (MADRS, a 10-item diagnostic questionnaire) were used to assess the severity of depression. The study was conducted on 153 patients (18–45 years old), six months or less postpartum with severe PPD (HAMD_17_ ≥ 26). Patients received zuranolone (30 mg) or placebo, once daily for 14 days, and were followed up through day 45. Zuranolone exerted a rapid (by day 3), clinically meaningful, and sustained response as compared to placebo. There was a significantly greater reduction from the baseline in the HAMD_17_ total score with zuranolone in comparison to placebo at day 15 through day 45. HAMD_17_ remission at day 15 was 45% in those receiving zuranolone, while it was 23% in the placebo group. There was also a sustained larger reduction from the baseline for the MADRS score with zuranolone at day 15. Anxiety symptoms, assessed by the HAMD_17_ anxiety/somatization subscale and the Edinburgh Postnatal Depression Scale (EPDS) anxiety subscale, demonstrated a significant improvement with zuranolone at days 3 through 45 compared to placebo. Rates of concurrent remission of depressive and anxiety symptoms were higher with zuranolone in comparison to placebo at days 3, 15, and 45. Furthermore, the rate of sustained concurrent remission at days 15 and 45 was also higher with zuranolone. In addition, zuranolone had beneficial effects on insomnia symptoms in PPD patients [81,82].

The SKYLARK study (217-PPD-301) was an additional phase 3, randomized, double-blind, placebo-controlled trial assessing the efficacy and safety of 50 mg zuranolone compared to placebo in women with severe PPD. The study was conducted on 196 patients (18–45 years old) with severe PPD (HAMD_17_ ≥ 26). Patients received zuranolone or placebo once nightly for 14 days, and then were followed up for an additional 4 weeks. By analogy to the results from the ROBIN trial, the antidepressant effects of zuranolone were rapid, starting at day 3, with a median time to first HAMD_17_ response of 9 days in the zuranolone group as opposed to 43 days in the placebo group. A significant improvement in depressive symptoms was also observed on days 28 and 45. The HAMD_17_ remission rate was greater for zuranolone compared with placebo at day 45 (44.0% vs. 29.4%). Patient-reported outcomes as measured by the EPDS and Patient Health Questionnaire-9 correlated well with the overall HAMD_17_ score. A total of 75.5% of patients participating in the SKYLARK study had moderate to severe anxiety (HAMA score ≥ 20). Improvements in anxiety at day 15, as assessed by a change from the baseline (CFB) in the HAMA score, were significantly greater in the zuranolone group compared with the placebo group. Overall, a significantly greater proportion of women receiving zuranolone achieved concurrent remission of depressive and anxiety symptoms compared with those receiving placebo at day 3 (18.9% versus 2.7%), day 15 (40.5% versus. 19.2%), and day 45 (52.1% versus 23.2%). Beneficial effects of zuranolone on insomnia were also observed. The Clinical Global Impression-Improvement (CGI-I) score at day 15 was also markedly higher in the zuranolone group as compared with placebo, indicating improvements across domains of patient quality of life [83].

Zuranolone was generally well tolerated. Both in the ROBIN and SKYLARK studies, the most common treatment-emergent adverse events (≥5% and greater than in the placebo group) in zuranolone-treated patients were somnolence, dizziness, diarrhea, fatigue, and urinary tract and upper respiratory tract infections. No evidence of withdrawal symptoms or increased suicidal ideation or behavior were identified [81,82].

Integrated functional health and well-being data, as assessed using the 36-Item Short Form Health Survey (SF-36), from three MDD trials (201B, MOUNTAIN, and WATERFALL) and one PPD trial (ROBIN), compared zuranolone (30 and 50 mg) with placebo at day 15 and day 42. SF-36 includes scales for physical functioning, social functioning, role limitations due to physical or emotional problems, mental health, energy, pain, and overall health perception. Participants treated with zuranolone showed early (day 15), clinically meaningful improvements in the analyzed parameters related to mental well-being: vitality, social functioning, role limitations due to emotional problems, and mental health. Importantly, patients also demonstrated improvements in the vitality domain, which is an indicator of improvement in energy, fatigue, and subjective well-being, as early as on day 15. This contrasts with SSRI treatments, where up to 23% of patients can experience reduced energy as the treatment-emergent side effect. Treatment with zuranolone and the time to response appeared to be associated with a return to normative levels of functioning and health-related quality of life. The results of this integrated analysis suggest that zuranolone may help address the unmet need for a fast-acting treatment that can potentially improve mental health, functioning, and well-being for patients with MDD and PPD [84,85].

Recently the group of Meltzer-Body [86], using data from randomized controlled trials, indirectly compared the effectiveness of zuranolone and SSRIs (fluoxetine, sertraline, paroxetine, citalopram, and escitalopram) in reducing depressive symptoms in patients with PPD. The effects of the drugs were assessed by CFB in EPDS and HAMD_17_ scores. A larger EPDS CFB was observed in zuranolone-treated patients in comparison to SSRI-treated subjects from day 15 onward. Patients treated with zuranolone exhibited a 4.22-point larger reduction in EPDS compared to placebo groups, and a 7.43-point larger reduction at day 45. It should be emphasized that, according to the investigators, “the results of this meta-analysis should be regarded with caution as the overall quality of evidence is low”.

In a randomized, phase 1, double-blind, active- and placebo-controlled, four-treatment, four-period crossover study, the effect of zuranolone (50 and 100 mg) on simulated driving performance was examined. In this study, the SDLP (a measure of lane position control) and additional driving performance assessments, such as lane exceedance, excessive speed count, excessive cornering speed threshold, and total number of collisions, were evaluated. One group of participants received 50 mg of zuranolone or placebo once nightly on days 1–7, while another group received zuranolone 50 mg or placebo on days 1–6 and then zuranolone 100 mg or placebo on day 7. Reduction in driving performance was typically observed after the first dose of zuranolone 50 mg. It decreased in magnitude on day 8 after repeated dosing and increased in magnitude on day 8 following administration of zuranolone 100 mg. The most frequently reported treatment-emergent adverse events occurring in ≥10% of participants who received 50 mg of zuranolone on days 1–7 were dizziness, somnolence, fatigue, tremor, headache, asthenia, nausea, insomnia, speech disorder, and constipation [87].

It is well known that the safety of antidepressants during lactation is of a high importance for women with a depressive disorder who choose to breastfeed. The cessation of breastfeeding was required in the clinical studies referenced above [81,82,83].. However, this aspect has not been thoroughly investigated. Taking into account a potential infant exposure to zuranolone via excretion into breast milk, Deligiannidis and coworkers assessed the extent of transfer of five 30 mg drug doses into the breastmilk of 15 healthy, non-pregnant, lactating adult females using RID (relative infant dose) values. RID is the weight-adjusted proportion of the maternal dose consumed by the infant in breast milk over a 24-h period. The RID at day 5 of 30 mg dose was 0.357%. The estimated mean RID of once-daily administration of 50 mg of zuranolone for 14 days was 0.74% and 0.98% with daily milk intake of 150 and 200 mg/kg per day, respectively. These RIDs are below 1% and well below the 10% RID threshold generally considered compatible with breastfeeding [88]. As this was a single study conducted on a small group of participants, additional studies are required to access whether treatment with zuranolone by breast feeding patients may affect the health condition of their infants.

## 5. Concluding Remarks

PPD is a significant health problem affecting not only mothers but also their children and children’s fathers. The currently used basic pharmacological treatment, including SSRIs, is often not effective. Neuroactive steroids offer several advantages over traditional antidepressants, including rapid onset, unique mechanism of action, and lack of tolerance upon repeated use. Zuranolone, the oral medication, represents a promising advancement in the neurosteroid-based treatment for PPD. Compared to brexanolone, it offers an easier and well-tolerated treatment option. As clinical trials on zuranolone conducted so far have been short-term ones with relatively small sample sizes, there is an urgent need for large-scale clinical studies. Moreover, a high under-representation in clinical trials on zuranolone of several ethnic groups (Table 2) constitutes a significant limitation in the comprehensive assessment of the efficacy and safety of treatment with zuranolone and, as highlighted by Swieczkowski and coworkers [89], clearly indicates the need to include ethnic minorities in future clinical trials.

## Figures and Tables

**Table 1:** FDA status of antidepressant drugs used for PPD treatment.

Drug	FDA Approval for PPD Treatment/Year of Approval	Other Indications Approved by FDA
Neuroactive steroids		None [44,45]
-Brexanolone [44]	YES/2019	
-Zuranolone [45]	YES/2023	
SSRIs [46]	NO	Major depressive disorder, generalized anxiety disorder, obsessive-compulsive disorder, panic disorder, post-traumatic stress disorder, social anxiety disorder, premenstrual dysphoric disorder, bulimia nervosa
SNRIs [47]	NO	Major depressive disorder, generalized anxiety disorder, social anxiety disorder, panic disorder, diabetic peripheral neuropathic pain, fibromyalgia, chronic musculoskeletal pain
TCAs [48]	NO	Major depressive disorder, obsessive-compulsive disorder (clomipramine)

**Table 2:** Characteristics of the population participating in clinical trials of zuranolone in PPD.

Reference	Treatment	Participants, No.	Ethnicity, No. (%)		Race, No. (%)			Change from Baseline in HAM-D Score	Treatment-Emergent Adverse Events No. (%)
			Hispanic or Latino	Not Hispanic or Latino	African American	White	Other ^a^		
[83]	zuranolone 30 mg	76	16 (21)	60 (79)	31 (41)	44 (58)	1 (1)	−17.8 points	47 (60)
	placebo	74	18 (24)	56 (76)	31 (42)	40 (54)	3 (4)	−13.6 points	38 (52)
[84]	zuranolone 50 mg	98	33 (33.7)	65 (66.3)	25 (25.5)	68 (69.4)	5 (5.1)	−15.6 points	65 (66.3)
	placebo	98	42 (42.9)	56 (57.1)	18 (18.4)	69 (70.4)	11 (11.2)	−11.6 points	52 (53.1)

^a^ Other includes Asian, Native Hawaiian or Other Pacific Islander, and more than 1 race.

## Data Availability

The original contributions presented in the study are included in the article; further inquiries can be directed to the corresponding author.

## Abbreviations

The following abbreviations are used in this manuscript:

AUC	Area under curve
CFB	Change from baseline
CGI-I	The Clinical Global Impression-Improvement
CL/F	Apparent clearance
Cmax	Maximum plasma concentration
DSM-5	Diagnostic and Statistical Manual of Mental Disorders—Fifth Edition
Emax	Maximum efficacy
EPDS	Edinburgh Postnatal Depression Scale
FDA	Food and Drug Administration
GABA	Gamma aminobutyric acid
GABAARs	GABAA receptors
HAMD17	17-item Hamilton Rating Scale for Depression
HAMD	Hamilton Depression Rating Scale
IP	Intraperitoneally
MAD	Multiple ascending dose
MADRS	Montgomery–Åsberg Depression Rating Scale
MDD	Major depressive disorder
NAS	Neuroactive steroid
PAMs	Positive allosteric modulators
PO	Per os
PPD	Postpartum depression
RID	Relative infant dose
SAD	Single ascending dose
SDLP	Standard deviation of lateral position
SF-36	36-Item Short Form Health Survey
SNRIs	Serotonin and noradrenaline reuptake inhibitors
SSRIs	Selective serotonin reuptake inhibitors
t1/2	Half-life time
TCAs	Tricyclic antidepressants
tmax	Time to reach maximum concentration
